# Can the post-ruminal urea release impact liver metabolism, and nutritional status of beef cows at late gestation?

**DOI:** 10.1371/journal.pone.0293216

**Published:** 2023-10-19

**Authors:** Marta M. Santos, Thaís C. Costa, Tiago A. O. Mendes, Luana L. Dutra, Davi N. L. Silva, Renato D. Araújo, Nick V. L. Serão, Luciana N. Rennó, Yamê F. R. S. Silva, Edenio Detmann, Javier Martín-Tereso, Isabela P. Carvalho, Mateus P. Gionbelli, Marcio S. Duarte

**Affiliations:** 1 Department of Animal Science, Universidade Federal de Viçosa, Viçosa, MG, Brazil; 2 Muscle Biology, and Nutrigenomics Laboratory, Universidade Federal de Viçosa, Viçosa, MG, Brazil; 3 Department of Animal Science, Universidade Federal de Lavras, Lavras, MG, Brazil; 4 Department of Biochemistry, and Molecular Biology, Universidade Federal de Viçosa, Viçosa, MG, Brazil; 5 StatsGaze Data Science Solutions, Liverpool, NY, United States of America; 6 Trouw Nutrition Research & Development, Amersfoort, The Netherlands; 7 Department of Animal Biosciences, University of Guelph, Guelph, ON, Canada; IZ: Instituto de Zootecnia, BRAZIL

## Abstract

We aimed to evaluate the effects of post-ruminal supply of urea (PRU) on nutritional status, and liver metabolism of pregnant beef cows during late gestation. Twenty-four Brahman dams, pregnant from a single sire, and weighing 545 kg ± 23 kg were confined into individual pens at 174 ± 23 d of gestation, and randomly assigned into one of two dietary treatments up to 270 d of gestation: Control (CON, n = 12), consisting of a basal diet supplemented with conventional urea, where the cows were fed with diets containing 13.5 g conventional urea per kg dry matter; and PRU (PRU, n = 12), consisting of a basal diet supplemented with a urea coated to extensively prevent ruminal degradation while being intestinally digestible, where the cows were fed with diets containing 14,8 g urea protected from ruminal degradation per kg dry matter. Post-ruminal supply of urea reduced the urine levels of 3-methylhistidine (*P* = 0.02). There were no differences between treatments for dry matter intake (DMI; *P* = 0.76), total digestible nutrient (TDN) intake (*P* = 0.30), and in the body composition variables, such as, subcutaneous fat thickness (SFT; *P* = 0.72), and rib eye area (REA; *P* = 0.85). In addition, there were no differences between treatments for serum levels of glucose (*P* = 0.87), and serum levels of glucogenic (*P* = 0.28), ketogenic (*P* = 0.72), glucogenic, and ketogenic (*P* = 0.45) amino acids, neither for urea in urine (*P* = 0.51) as well as urea serum (*P* = 0.30). One the other hand, enriched pathways were differentiated related to carbohydrate digestion, and absorption, glycolysis, pyruvate metabolism, oxidative phosphorylation, pentose phosphate pathway, and biosynthesis of amino acids of the exclusively expressed proteins in PRU cows. Shifting urea supply from the rumen to post-ruminal compartments decreases muscle catabolism in cows during late gestation. Our findings indicate that post-ruminal urea supplementation for beef cows at late gestation may improve the energy metabolism to support maternal demands. In addition, the post-ruminal urea release seems to be able to trigger pathways to counterbalance the oxidative stress associated to the increase liver metabolic rate.

## Introduction

In bovines, more than 50% of placental energy demand during the last third of gestation is supplied by amino acids [1]. The potential of fetal growth based on maternal fat reserves is low [2] due to the limited transport of fatty acids, and ketone bodies through the placenta [3]. Studies have shown changes in plasmatic regulators of protein catabolism, and nitrogen losses of ewes during the last third of gestation, suggesting nitrogen mobilization from maternal lean tissue [4,5] as an attempt to meet fetal requirements.

However, nitrogen (N) metabolism in ruminants also involve other high-priority functions, such as nitrogen recycling to the gastrointestinal tract [6]. Normally, urea synthesis is performed from cytoplasmatic, and mitochondrial N pools, where N atoms can be absorbed from both ammonia, and amino acids [7]. It has been demonstrated that urea supplementation can improve the efficiency of the utilization of amino acids in body anabolism [8]. Recently, it has been shown that supplementation with post-ruminal urea (PRU) release provides greater efficiency in nitrogen usage in rumen, and animal body due to greater, and more stable ruminal N recycling, greater uptake of recycled N by rumen microorganisms, decreased urinary-N loss, and improvement in fiber digestion when compared to the classical releasing of urea as pulse dose in the rumen [9,10]. In addition, the absorption of urea in the small intestine can avoid an overload of ammonia in the liver, which occurs after ruminal degradation of dietary urea [10].

Late gestation is a critical period for a pregnant dam [11] as the nutritional requirements are enhanced due to an increase in growth rate of the fetus, while the dry matter intake capacity by the cows is limited [12]. Thus, moving the release of dietary urea from the rumen to the intestinal tract may be strategically used to overcome the challenging nutritional conditions of pregnant cows at late gestation. Therefore, we aimed to evaluate the impact of shifting urea release from rumen to the intestinal tract at late gestation on liver metabolism, and nutritional status of pregnant cows at late gestation.

## Materials and methods

### Animal ethics

All experimental procedures were approved by the Ethical Committee on Animal Use of the Department of Animal Science at *Universidade Federal de Viçosa*, *Minas Gerais*, Brazil (protocol 33/2020).

### Animals and experimental diets

Twenty-four Brahman dams, pregnant from a single sire, weighing 545 kg ± 23 kg, and 174 ± 23 d of gestation were confined at into individual pens with an area of 12 m^2^, and provided with bunkers, and water bowls. The twenty-four cows used in the trial were pulled from a herd that was submitted to a reproductive protocol using three attempts of fixed-time artificial insemination. Consequently, a total of three groups of cows according to days of gestation was obtained. Thus, at the beginning of the trial the cows within the gestational time groups were evenly allocated to the two experimental treatments. However, the endpoint of the trial was 270 days of gestation to all cows and thus, the gestation group (1 to 3) was considered as a fixed effect in the statistical model. All cows were initially submitted to an adaptation period of 10 d receiving water *ad libitum*, and diet containing corn silage, ground corn, soybean meal, and mineral mixture. After the adaptation period, the fetal sex was determined via ultrasound on every cow, and within each pregnancy group, and fetal sex combination, cows were randomly assigned into two experimental treatments up to 270 d of gestation: Control (CON, n = 12), where cows were fed a basal diet containing conventional urea; and PRU (PRU, n = 12), where cows were fed a basal diet containing urea protected from ruminal degradation to partially be released in the small intestine (Trouw Nutrition, Netherlands) (Table 1). The post-ruminal urea (PRU) product was subjected to an in vitro evaluation to estimate ruminal protection rate [13]. Approximately 35% of this urea is released into the rumen, and the remainder (65%) is released into the post-ruminal compartments. The dry matter intake was adjusted weekly to meet 100% maintenance requirements [14] based on the body weight, and gestational age.

**Table 1:** Ingredients, and chemical composition of the experimental diets.

Ingredients	CON (g/kg)	PRU (g/kg)
Corn silage^a^	700.0	700.0
Ground Corn ^a^	227.5	226.2
Soybean meal^a^	45.0	45.0
Feed-grade urea^a^	13.5	-
Rumen protected urea^a^	-	14.8
Mineral mixture^a^^,^^b^	14.0	14.0
Chemical composition	g/kg	
Dry matter^c^	509	509
Crude protein^a^	116	119
Ether extract^a^	31	31
Neutral detergent fiber^a^	364	364
Non-fibrous carbohydrates^a^	430	427
Crude ash^a^	59	59

CON = Control treatment; PRU = post-ruminal releasing of urea.

^a^g/kg dry matter.

^b^Composition per kg: 76 g of calcium, 65.6 g of phosphorus, 34 g of sulfur, 14.4 g of magnesium, 61 g of sodium, 890 mg of copper, 48.4 mg of cobalt, 61 mg of iodine, 740 mg of manganese, 3340 mg of zinc, 16.5 mg of selenium.

^c^g/kg as fed.

### Animal performance data, and sample collection

To evaluate the nutritional characteristics of the diet, samples of roughage, and concentrate were collected weekly, and stored at -20°C for further chemical analysis. To evaluate the total digestibility, the experiment was divided into three periods of 28 d each, and approximately 200 g of feces were sampled, via manual collection directly from the rectum, on the 17 d at 6 am, 18 d at 12 pm, and 19 d (at 6 pm, three hours after second feeding) of each experimental period. Simultaneously to fecal sample collection, approximately 50 ml of urine was sampled by stimulated urination. Immediately after sampling, 5 ml of urine was filtered, diluted in 20 ml of sulfuric acid (0.036 N), and stored at -20°C. Upon the third urine collection (collected at 19 d of the experimental period), the first two samples (previously collected at the 17 d, and 18 d of the experimental period) were thawed, and the three samples were mixed, and stored -20°C for further analyses for urea, and 3 methyl-histidine.

On the 28 d of each experimental period, cows’ blood samples were collected in the morning via jugular venipuncture into vacuum tubes (BD Vacutainer, NJ, USA) containing heparin to evaluate the amino acid profile, tubes containing sodium fluoride to evaluate the concentration of glucose, and tubes containing gel, and clot activator to evaluate the concentration of urea. Immediately after collection, the samples were sent to a commercial laboratory (Viçosa Lab, Viçosa, Brazil). Simultaneously with the blood collection, carcass ultrasound images were collected by using a Aloka SSD500 with a 3.5 Mhz 18 cm linear probe to monitor the body condition score of the cows. Images were captured and analyzed by using the Biosoft ToolBox for Bovine (Biotronics, Ames, Iowa, USA).

On the 54 d of the feeding trial, liver biopsies were performed using specific needles (Tru-Cut biopsy needle, Care Fusion Corporation, San Diego, California, USA), according to the procedures previously described [15]. The area between the 11^th^ -12^th^ rib was initially cleaned with 70% ethanol, and the incision was performed 10 min after local anesthesia treatment (Lidocaine 2%). Liver samples (~ 30 mg) from the right lobe were collected. Immediately after collection, the samples were rinsed with phosphate saline buffer (pH = 7.4) and immediately snap-frozen in liquid nitrogen, and kept at -80°C for further analysis.

### Feed, and fecal samples chemical analysis

Samples of feeds, and feces were oven-dried (60°C), and ground in a Wiley mill model 3 (Thomas Scientific, NJ, USA) to pass through a 2- mm screen. After that, half of each ground sample was ground again to pass through a 1- mm screen. The fecal samples were pooled on an air-dry weight basis per day, and animal according to the periods of collection (equal weight for each collection time).

The samples ground to pass through a 1- mm screen sieve were used to perform the analyses of dry matter [16] (DM; method 934.01), crude protein [16] (CP; method 990.13), ether extract [16] (EE; method 920.39), ash [16] (method 942.05), neutral detergent fiber corrected for ash, and protein (NDFap; using a heat stable α-amylase omitting sodium sulfite; [17]; methods INCT-CA F-002/1; N-004/1, and M-002/1). The non-fibrous carbohydrates (NFC) were calculated as previously proposed [18]. In addition, the samples ground to pass a 2- mm screen sieve were incubated in quadruplicate in the rumen of two cows for 240 h using non-woven textile bags (100 g/m^2^) to quantify the indigestible NDF (iNDF) [19]. The fecal excretion of DM was estimated by using iNDF as an internal marker. We calculated the digestibility coefficients for the different feed components to assess the digestible energy intake, which was expressed in terms of total digestible nutrients (TDN).

### Analysis of blood, and urinary parameters

In urine samples the concentrations of urea (Atellica Solution™, Siemens Healthineers, Erlangen, Forchheim, Germany), and 3 methyl-histidine (HPLC, Hermes Pardini, Belo Horizonte, Minas Gerais, Brazil) were determined. In blood samples, analyses of glucose (Cobas C311 analyzer, Roche Diagnostics, Rotkreuz, Switzerland), urea (Cobas C311 analyzer, Roche Diagnostics, Rotkreuz, Switzerland), and the amino acid profile were performed, and the following serum amino acids were quantified: glutamic acid, alanine, glycine, methionine, valine, citrulline, ornithine, leucine, phenylalanine, tyrosine, and isoleucine (LC-MS/MS, Xevo™ TQD, Waters Corporation, Milford, Massachusetts, USA).

### Liver tissue protein extraction

Total proteins were extracted from 1 mg of tissue in a 1 mL of lysis buffer (7 M urea, 2 M thiourea, 4% 3-((3-cholamidopropyl) dimethylammonio)-1-propanesulfonate (CHAPS) detergent, 1% dithiothreitol (DTT), and 10 ul protease inhibitor cocktail (Sigma-Aldrich®, St. Louis, Missouri, USA), homogenized using a shaft-type homogenizer (LabGEN 125, Cole-Parmer, Bunker Hill, IL, USA), and then centrifuged at 10000 × g for 30 min at 4°C. The supernatant was collected, aliquoted, and stored at -80°C. Protein content was estimated by the Bradford Protein Assay (Bio-Rad, Hercules, CA, USA). After protein quantification, 50 μg of sample was transferred to 2.5 μL tuber containing 100 mM of DTT (1.4-dithiothreitol). The solution was then stirred, and placed in a thermal block at 60°C for 30 min. After reaching the room temperature, 2.5 μL of 300 mM iodoacetamide was added, for cysteine alkylation, and transferred to the dark at room temperature for 30 min. 10μL of trypsin (Promega Corporation, Madison, WI, USA) was added to an ammonium bicarbonate (Ambic) solution, vortexed, and digested overnight at 37°C. The samples were then dried using a SpeedVac^TM^ centrifuge (Thermo Fisher Scientific, Waltham, MA, USA), resuspended in 50 μL of 0.1% trifluoroacetic acid (TFA) solution prepared in H2O milliQ, and desalted using ZipTip^®^C18 (Merck Millipore, Billerica, MA, USA).

### Protein identification, and data processing

Protein identification, and quantification was performed in a NanoAquity high-performance liquid chromatographer (HPLC) coupled with a maXis 3G high-resolution Q-TOF mass spectrometer (Bruker Daltonics, Billerica, Massachusetts, USA). The raw data were processed with MaxQuant software (v. 1.6.3.3), with the parameters set to default values, considering the protein amino terminal acetylation, methionine oxidation as variable modification, and the fixed modification as carbamidomethylation of cysteine. The trypsin specificity was kept as the digestion mode, and the instrument selected was Bruker-QTOF, set to default, including the parameters of first (20 ppm), and main (10 ppm) search peptide tolerance. The label-free quantification (LFQ) mode was added, and at least two unique peptide ratios (min LFQ ratio count = 2) were considered. The bovine reference proteome was obtained from UniProt (ID: UP000009136) available in (www.uniprot.org, accessed on 23 March 2021). A total of 807 proteins were identified in the liver of cows.

Prior to normalization of the data, the processed proteomic abundance data were subjected to quality control. For this, samples (i.e., animals), and proteins identified as potential contaminants, only identified by site, and reverse sequence were removed from the dataset to avoid issues with the statistical analyses, and inferences. First, samples with less than 1% of the proteins identified were removed from the dataset. In this step, two samples (both from CON) were removed, resulting in a final dataset with 22 animals (10 from CON, and 12 from PRU) with proteomic data. Afterwards, PTNs represented in less than 10% of samples (i.e., in 2 or fewer samples) were removed. The final dataset included 382 proteins.

After quality control, the data were subjected to normalization of the library size. Normalizing factors were obtained for each protein using the Trimmed Mean of M-values method, via the *TMM package* [20] implemented in *R* (R Core Team, Vienna, Austria).

### Statistical analyses

#### Analyses of data of animal performance, nutritional, and physiological blood characteristics

Data from animal nutritional, and physiological blood parameters were analyzed according to the following model below:

$$y_{ijkl}=\mu +T_{i}+{GG}_{j}+{FS}_{k}+b_{1}{IW}_{ijkl}+e_{ijkl}$$ [Eq 1]

where *y*_*ijkl*_ is the observed data for the *l*^th^ cow; *μ* is the intercept; *T*_*i*_ is the fixed-effect of the *i*^th^ Treatment (*i* = 1, 2); *GG*_*j*_ is the fixed-effect of the *j*^th^ Gestation Group (*i* = 1 to 3); and *FS*_*k*_ is the fixed-effect of the *k*^th^ Sex of the Fetus (*k* = 1, 2); *b*_1_ is the partial regression coefficient for the fixed-effect covariate of initial body weight; *IW*_*ijkl*_ is the initial body weight of the cow at the beginning of the feeding period (in kg) of the *l*^th^ cow; and *e*_*ijkl*_ is the random error for the *l*^th^ cow, assuming $e_{ijkl}∼N(0,I\sigma_{e}^{2})$, where ***I*** is the identity matrix, and $\sigma_{e}^{2}$ the residual variance. The covariate of initial body weight of the cow at the beginning of the feeding period in Eq 1 was kept in the model for traits when highly significant (*P* < 10^−5^).

Prior to analyses, residuals were evaluated for distributional assumptions. Studentized residuals outside ± 3 SD were removed, one at a time, while simultaneously assessing their homogeneity across fixed-effects with visual inspection, and their normality with Shapiro-Wilk’s test (*P* > 0.05) [21]. After removal of outliers, all the data showed normality, and homogeneity of the residual, and were subjected to subsequent final analyses. All analyses were performed using the GLIMMIX, and UNIVARIATE procedures of SAS 9.4 (Statistical Analysis System Institute, Inc., Cary, NC, USA). Significances were declared at *P* < 0.05, and trends were discussed when 0.10 < *P* ≤ 0.05.

#### Models evaluated for proteomic analysis

Multiple distributions of the normalized data, and models were evaluated for each of the 382 proteins analyzed. In this step we evaluated Negative Binomial (NB) distributions assuming or not zero-inflated (ZI) data using a log-link function [22]. Therefore, two combinations were evaluated, one not assuming zero-inflation of the data (i.e., many samples without abundance for the protein analyzed), and referred to just as NB, and one assuming zero-inflation (ZINB).

For the NB model, the data were analyzed as:

$$ln\left(\frac{y_{ijkl}}{{LS}_{ijkl}}\right)=\mu +T_{i}+{GG}_{j}+{FS}_{k}$$ [Eq 2]

where $ln\left(\frac{y_{ijkl}}{{LS}_{ijkl}}\right)$ represents the natural logarithm of the normalized protein abundance analyzed adjusted for the normalized library size for the *l*^th^ animal, and all other terms have been defined in Eq 1.

When assuming ZI data, we used three models where the count (i.e., non-ZI) part of the model was the same as in Eq 2, and the ZI part was different. The first ZI model (ZINB_basic_) assumed only the intercept, as:

$$ln\left(\frac{y_{ijkl}}{{LS}_{ijkl}}\right)=\left\{ \begin{array}{l} \mu +T_{i}+{GG}_{j}+{FS}_{k}\;if\;y_{ijkl}>0\\ \mu_{ZI}\;if\;y_{ijkl}=0 \end{array}\right.$$ [Eq 3]

where *μ*_*ZI*_ represents the intercept of the ZI part of the model, and all other terms have been previously described in Eq 2. In ZINB_basic_, we assumed that over the presence of many zero in the data is only a function of the intercept.

In the other two models, Eq 3 was expanded to include the effects in Eq 1. First, only the effect of treatment was used (ZINB_TRT_):

$$ln\left(\frac{y_{ijkl}}{{LS}_{ijkl}}\right)=\left\{ \begin{array}{l} \mu +T_{i}+{GG}_{j}+{FS}_{k}\;if\;y_{ijkl}>0\\ \mu_{ZI}+T_{{ZI}_{i}}\;if\;y_{ijkl}=0 \end{array}\right.$$ [Eq 4]

where $T_{{ZI}_{i}}$ represents the *i*^th^ fixed effect of Treatment (*i* = 1,2) in the ZI part of the model; and all other terms have been previously described in Eqs 1 and 2. The ZI_TRT_ model was used to evaluate whether the presence of many zero in the data could also be explained by the maternal treatment. Finally, the last model assumed the same effects in both the non-ZI, and ZI parts of the model (ZINB_full_):

$$ln\left(\frac{y_{ijkl}}{{LS}_{ijkl}}\right)=\left\{ \begin{array}{l} \mu +T_{i}+{GG}_{j}+{FS}_{k}\;if\;y_{ijkl}>0\\ \mu_{ZI}+T_{{ZI}_{i}}+{GG}_{{ZI}_{j}}+{FS}_{{ZI}_{k}}\;if\;y_{ijkl}=0 \end{array}\right.$$ [Eq 5]

where ${GG}_{{ZI}_{j}}$ represents the *j*^th^ fixed-effect of Gestation Group (*j* = 1 to 3) in the ZI part of the model; ${FS}_{{ZI}_{k}}$ represents the *k*^th^ fixed-effect of Fetus Status (*k* = 1 to 3) in the ZI part of the model; and all other terms have been previously described in Eqs 2 to 4. With this, four models were evaluated for each protein. Analyses of all models were performed in SAS 9.4 (Statistical Analysis System Institute, Inc., Cary, NC, USA) with the GLIMMIX procedure.

#### Model selection and identification of differentially abundance proteins (DAPs)

The NB, ZINB_basic_, ZINB_TRT_, and ZINB_full_ models were compared within each protein analyzed. Only models that converged were evaluated. Model comparisons followed a nested strategy, from the simplest (NB) to the most complex (ZINB_full_) model using a likelihood ration test (LRT) to evaluate the inclusion of additional parameters into the model. That is, first, NB was compared to ZINB_basic_, and if the LRT was not significant (*P* > 0.05), then the NB model was used for the protein. However, if the LRT between NB, and ZINB_basic_ was significant (*P* > 0.05), results from the ZINB_basic_ were compared with the ZINB_TRT_. Likewise, if the LRT test between these were significant (*P* < 0.05), then the ZINB_TRT_ model was chosen, otherwise, ZINB_basic_ was used for the protein. Finally, if ZINB_TRT_ was selected in place of ZINB_basic_, it was then compared with ZINB_full_ to evaluate the best model for the protein analyzed. This process was used for each of the 382 proteins analyzed, and hence, the final results include different models, according to the protein.

After models were selected for each protein, effects in the model were deemed significant after adjusting *P*-values for multiple comparisons using False-Discovery Rate (FDR) [23] as *q*-values. Differentially abundant proteins (DAP) were identified at *q*-value < 0.05. The calculation of *q*-values was obtained with the *qvalue* package [24] in *R* version 3.6.3 [25] in *RStudio* version 3.0.386 [26].

#### Gene ontology (GO) and signaling pathways (KEGG) analyses

The protein-protein interaction network, Kyoto Encyclopedia of Genes, Genomes (KEGG), and Gene Ontology (GO) enrichment analyses were performed by String 11.0 (string-db.org). The interaction network of the exclusive proteins from the treatments CON, PRU, and the DAPs were obtained using the available interaction map from *Bos taurus* with the default option (medium confidence given by score of 0.4) [27]. The functional classification of the GOs and KEGG signaling pathways were deemed significant at an FDR-adjusted *P*-value (*P*_FDR_) < 0.05 based on Benjamini-Hochberg’s method [28].

## Results

### Animal performance, digestibility, and blood parameters

There were no differences between treatments for dry matter intake (DMI; *P* = 0.99), total digestible nutrient (TDN) intake (*P* = 0.99). The digestibility variables, dry matter (*P* = 0.58), crude protein (*P* = 0.51), ether extract (*P* = 0.73), non-fiber carbohydrates (*P* = 0.68), neutral detergent fibre corrected for ash, and protein (*P* = 0.95) did not differ between treatments. In addition, there were no differences in body composition variables, such as subcutaneous fat thickness (SFT; *P* = 0.72), and rib eye area (REA; *P* = 0.85). The concentrations of urea in urine (*P* = 0.51), and serum (*P* = 0.30) were also not different among treatments. Similarly, no changes in serum levels of glucose (*P* = 0.87), and in glucogenic (*P* = 0.28), ketogenic (*P* = 0.72), glucogenic, and ketogenic (*P* = 0.45) amino acids were observed among treatments. Similarly, the maternal treatment did not influence (*P* = 0.59) the birth weight of the progeny. On the other hand, the levels of 3 methyl-histidine, in mmol/mol creatine, were higher (*P* = 0.02) in CON. Results are presented in Table 2.

**Table 2:** Intake, digestibility, performance, urine, and serum parameters from treatment PRU, and CON.

Item	CON	PRU	*P*-value
*Intake*			
Dry matter intake (kg/day) Total digestible nutrient intake (kg/day)	5.9 ± 0.28 4.0 ± 0.19	5.9 ± 0.27 4.0 ± 0.18	0.99 0.95
*In vivo digestibility (%)*			
Dry matter	65.7 ± 0.48	66.1 ± 0.44	0.58
Crude protein Ether extract Non-fiber carbohydrates NDFap^a^	62.5 ± 0.97 81.6 ± 0.73 78.3 ± 1.25 59.8 ± 0.56	61.6 ± 0.92 81.3 ± 0.69 77.6 ± 1.18 59.9 ± 0.53	0.51 0.73 0.68 0.95
*Performance*			
Rib eye area (cm^2^)	62.3 ± 3.79	63.2 ± 3.60	0.85
Rib fat thickness (mm)	3.5 ± 0.63	3.8 ± 0.59	0.72
Average daily gain (kg/day)	0.38 ± 0.1	0.32 ± 0.09	0.66
Final body weight (kg)	589 ± 6.86	583 ± 6.44	0.47
*Serum parameters*			
Glucogenic amino acids^b^ (μmol/L) Ketogenic amino acids^c^(μmol/L) Glucogenic, and ketogenic amino acids^d^ (μmol/L) Glucose (mg/dL) Urea (mg/dL)	163.0 ± 5.59 19.7 ± 1.02 39.7 ± 3.19 60.4 ± 2.42 28.1 ± 1.44	170.9 ± 5.31 20.1 ± 0.98 42.8 ± 3.03 60.9 ± 2.29 26.2 ± 1.36	0.28 0.72 0.45 0.87 0.30
*Urine parameters*			
Urea (mg/dL) 3 methylhistidine (mmol/mol creatinine)	1157.5 ± 143.3 8.76 ± 0.50	1036.4 ± 135.8 7.19 ± 0.48	0.51 0.02
*Offspring’s performance (kg)* Birth weight	33.1 ± 1.64	34.2 ± 1.60	0.59

CON = Control treatment; PRU = Post-ruminal releasing of urea.

^a^Neutral detergent fibre corrected for ash, and protein.

^b^Glutamic acid, alanine, glycine, methionine, valine, citrulline, and ornithine.

^c^Leucine.

^d^Phenylalanine, tyrosine, and isoleucine.

### Proteomic profile of hepatic tissue

To identify the differentially abundant proteins (DAPs), the 382 proteins were tested. From these, 19 DAPs were identified (*q*-value < 0.05) between treatments, seven, and 12 proteins showing greater, and lower abundance in the PRU group compared to CON (Table 3). Among them, S-adenosylmethionine synthase isoform type 1 (MAT1A), methyl malonyl-CoA isomerase (MMUT), and persulfide dioxygenase (ETHE1) were more abundant in PRU compared to CON cows. On the other hand, the proteins apoptosis inducing factor mitochondrial associated (AIFM1), galectin-1 (LGALS1), and e lipoamide acyltransferase component of branched-chain alpha-keto acid dehydrogenase complex mitochondrial (DBT) were less abundant in PRU compared to CON cows.

**Table 3:** Differentially abundant proteins (DAPs) in the hepatic tissue of the cow.

Accession	Protein name	Gene name	*q*-value	ln (FC)^a^
				[95%CI]^b^
Q1LZH1:G5E6I5	Mitochondrial amidoxime reducing component	*MTARC*	1.65E-271	0.49
A0A3Q1LUG9;F1MKS3	Thioredoxin domain-containing protein 5	*TXNDC5*	1.65E-271	0.58
F1N5J8;A0A3Q1LNW7	2,4-dienoyl-CoA reductase 1	*DECR1*	1.65E-271	1.03
Q3T094	Persulfide dioxygenase	*ETHE1*	1.92E-139	0.26
Q9GK13;A0A3S5ZPN0	Methylmalonyl-CoA isomerase	*MMUT*	2.98E-134	0.24
A0A3Q1M909;P54149;A0A3Q1N5G3	Mitochondrial peptide methionine sulfoxide reductase	*MSRA*	3.51E-70	0.17
A0A3Q1NCY0;G5E5U7;Q2KJC6;A7E3T7;A0A3Q1MZ37	S-adenosylmethionine synthase isoform type-1	*MAT1A*	2.57E-44	0.16
F1MYG5;E1B8N6;A7YY47;A0A3Q1LNG7; F1MJI7	Lamin	*LMN*	1.65E-271	-1.53
A0A3Q1MTB8;Q28034;A0A3Q1MKJ7	Glucosidase 2 subunit beta	*PRKCSH*	1.65E-271	-1.48
F6PRB5;A0A3Q1LS67	Enoyl-CoA hydratase 1	*ECH1*	1.65E-271	-1.15
Q5I597;A0A452DHV7;A0A3Q1NDS5	Betaine—homocysteine S-methyltransferase	*BHMT*	1.65E-271	-0.35
P11116	Galectin-1	*LGALS1*	2.06E-264	-0.36
Q0VCU1;F1MS05;A0A3Q1MHC4	Cytoplasmic aconitate hydratase	*ACO1*	3.92E-155	-0.18
F1N5Q0;E1BJ78;A0A3Q1MP36;A0A3Q1M941; E1BIS9	Sulfotransferase	*SULT*	8.00E-148	-0.24
E1BJA2	Apoptosis inducing factor mitochondria associated	*AIFM1*	1.19E-43	-0.11
A7MBI6	Glyoxalase domain-containing protein 4	*GLOD4*	1.09E-33	-0.11
P68530	Cytochrome c oxidase subunit 2	*MT-CO2*	3.06E-18	-0.08
A0A3Q1MC79;F1MYV0;A0A3Q1LW09	Solute carrier organic anion transporter family member 1B3	*SLCO1B3*	1.17E-15	-0.07
A0A3Q1MED4;P11181	Lipoamide acyltransferase	*DBT*	4.42E-09	-0.08

CON = Control treatment; PRU = Post-ruminal supplementation with urea; ^a^Negative, and positive natural log fold changes [ln(FC)] indicate, respectively, lower, and greater protein abundance in the treatment PRU compared to CON; ^b^95% confidence interval.

### Functional analysis of the differentially abundant proteins

The GO can be classified as biological processes, molecular function, and cellular components. The DAPs network showed biological processes related to sulfur amino acid metabolism (GO:0000096; *P*_FDR_ = 0.02), and nitrate metabolic process (GO:0042126; *P*_FDR_ = 0.02) upregulated in PRU compared to CON cows. The enriched molecular function related to catalytic activity (GO:0003824; *P*_FDR_ < 0.03), and binding proteins (GO:0005488; *P*_FDR_ < 0.03), showed up, and down-regulation in PRU compared to CON cows, respectively. Regarding the cellular components, mitochondrion term (GO:0005739; *P*_FDR_ < 0.01) was upregulated in PRU compared to CON cows. The KEGG signaling pathways analysis identified the upregulation of fatty acids biosynthesis (WP1020; *P*_FDR_ = 0.03), and metabolism of vitamin B12 (WP3193; *P*_FDR_ = 0.04) in PRU compared to CON cows. Conversely, the apoptosis (bta04210; *P*_FDR_ < 0.01), and FAS (WP1019; *P*_FDR_ < 0.01) pathways were downregulated in PRU compared to CON cows.

### Functional analysis of the exclusive protein

We found 280 and 83 exclusive proteins in the treatments PRU and CON, respectively. The protein-protein interaction network of proteins found to be exclusive in each treatment were significant (PRU: *P*_FDR_ < 1.0e-16; CON: *P*_FDR_ = 2.1e-7), indicating that the proteins are biologically connected.Due to the greater number of proteins found to be exclusively present in each treatment, we identified a variety of proteins which participate in pathways involved in nitrogen and energy metabolism that will be further emphasized in discussion section. The analysis of KEGG pathways from the exclusive proteins in treatment PRU indicate pathways of interest related to pentose phosphate pathway (PGD), pyruvate metabolism (DLAT, LDHA), glycolysis (ALDOA, PGAM1, PKLR), carbohydrate digestion, and absorption (ATP1A1, ATP1A4, ATP1A3, ATP1A2), cysteine, and methionine metabolism (MTAP, ADI1), and oxidative phosphorylation (NDUFA8, NDUFAB1, NDUFS1, NDUFV2, UQCRB, UQCRH, UQCRC1, COX72A, COX6B1, COX5B, MGC148714, ATP5D, ATP5F1, ATP5J, ATP5J2) (Table 4). For the proteins exclusively found in CON cows, KEGG analysis identified enriched pathways related to retinol (CYP1A2, CYP3A24, LOC100138004, UGT1A1), and tryptophan metabolism (CYP1A2, DLD, GCDH) (Table 4).

**Table 4:** Enriched metabolic pathways of the exclusive proteins from treatment PRU, and CON.

KEGG ID	Description	*P*_FDR_^a^	Protein symbols^b^
*Treatment PRU*			
bta00190	Oxidate phosphorylation	2.15E-09	ATP5D, UQCRB, NDUFA8, COX7A2, COX6B1, NDUFAB1, ATP5F1, PPA1, UQCRH, CYC, MGC148714, COX5B, UQCRC1, NDUFS1, ATP5J, ATP5J2, NDUFV2
bta00030	Pentose phosphate pathway	0.0021	DERA, TALDO1, ALDOA, PGD, GLYCTK
bta00280	Valine, leucine, and isoleucine degradation	0.0424	AOX1, HMGCS1, BCKDHA, ALDH1B1
bta04975	Cholesterol metabolism	0.0021	LIPA, CYP27A1, VAPA, VAPB, NPC2, ABCB11
bta00010	Glycolysis / gluconeogenesis	0.0045	LDHA, DLAT, ALDOA, ALDH1B1, PGAM1, PKLR
bta04973	Carbohydrate digestion, and absortion	0.0302	ATP1A1, ATP1A4, ATP1A3, ATP1A2
bta00270	Cysteine, and methionine metabolism	0.0424	ADI1, LDHA, MTAP, GCLC
bta01230	Biosynthesis of amino acids	0.0332	ADI1, LDHA, MTAP, GCLC
bta00620	Pyruvate metabolism	0.0220	LDHA, DLAT, ALDH1B1, PKLR
bta04141	Protein processing in endoplasmic reticulum	0.0077	CRYAB, SSR1, LMAN2, HYOU1, SAR1A, BCAP31, SAR1B, HSPA6, RPN2
bta03050	Proteasome	0.0021	PSMB2, PSMA7, PSMB1, PSMA4, PSMA8, PSME1
bta03010	Ribosome	0.000022	RPS17, RPS28, RPL23, RPS9, RPL12, RPS2, RPS19, RPS5, RPS14, RPS24
bta04145	Phagosome	0.0021	CTSL1, LOC100141266, TUBA4A, ACTG1, MBL2, SEC22B, RAB7A, CGN1, CTSV, LAMP1
bta00480	Glutathione metabolism	0.0157	GSR, MGST3, PGD, GSTA5, GCLC
bta04142	Lysosome	1.22E-9	GUSB, CTSL1, AP1B1, MAN2B1, NAGA, SCARB2, ASAH1, LIPA, HEXA, GBA, TPP1, CTSV, CTSZ, NPC2, LAMP1, HEXB, LBMN
*Treatment CON*			
bta00380	Tryptophan metabolism	0.0209	CYP1A2, DLD, GCDH
bta00830	Retinol metabolism	0.0036	CYP1A2, CYP3A24, LOC100138004, UGT1A1

CON = Control treatment; PRU = post-ruminal supplementation with urea; ^a^False Discovery Rate (FDR)-adjusted *P*-value; ^b^Proteins corresponding to the connected proteins in the protein-protein interaction network.

## Discussion

**Fig 1:**
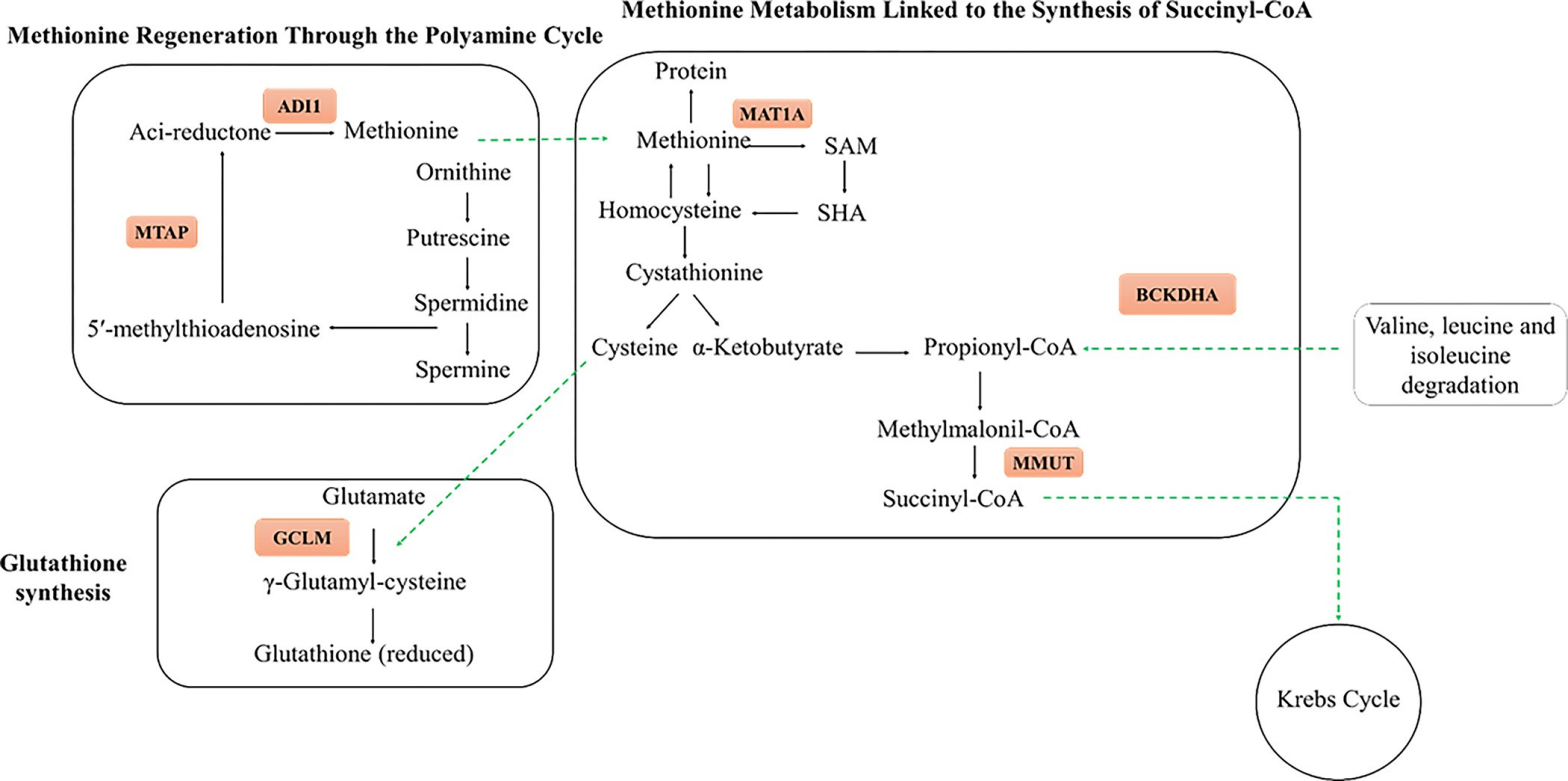
Enriched energy protein pathways of the exclusive proteins from treatment PRU. Summary of the protein metabolism pathways influenced by the experimental treatments. Proteins inside the orange square are positively regulated in treatment PRU (post-ruminal releasing of urea) compared to CON. ADI1: Acireductone dioxygenase 1; MTAP: Methylthioadenosine phosphorylase; GCLM: γ-glutamylcysteine synthase; BCKDHA: Branched chain keto acid dehydrogenase E1 subunit alpha; MMUT: Methylmalonyl- CoA isomerase; MAT1A: S-adenosylmethionine synthase isoform type-1.

Studies evaluating the post-ruminal urea supplementation have recently emerged [9,10,13], however, the effects of the post-ruminal supply of urea in cows during late gestation are not fully understood. Therefore, in the present study we evaluated the impact of shifting urea release from rumen to the intestinal tract at late gestation on liver metabolism, and nutritional status of pregnant cows at late gestation. To further explore the consequences of PRU supplementation in the metabolism, we employed a shot-gun proteomic approach, and a pathway analysis to identify the possible biological changes in the liver. We were able to identify the enhancement of S-adenosylmethionine synthase isoform type 1 (MAT1A) in PRU compared to CON cows. The protein MAT1A is involved in methionine metabolism, catalyzing the conversion of methionine into S-adenosylmethionine (SAM), which is demanded for the biosynthesis of polyamines, and glutathione (GSH) [29] (Fig 1). The proteins methylthioadenosine phosphorylase (MTAP), and acireductone dioxygenase 1 (ADI1), found to be exclusively expressed in PRU cows, are involved in methionine regeneration from the polyamines [30]. The polyamines (putrescine, spermidine, and spermine) are key regulators of placental angiogenesis, and embryo development [31]. During the process of methionine regeneration from polyamines, the sulfur of 5’-methylthioadenosine (MTA) is recycled, synthesizing acireductone through the action of the enzyme MTAP [32]. The acireductone is then converted into methionine through the reaction catalyzed by ADI1 [32]. Taken together, the proteins exclusively expressed in PRU cows indicate an enriched pathway of methionine regeneration from polyamines. Kwon [33] reported a greater concentration of polyamines in the first third of gestation in ovine due to the intensive placental development, accompanied by its reduction as the pregnancy advances. Providing greater availability of protein in the intestines contributed to an increase in placenta angiogenesis, and blood flow during gestation through nitric oxide [34,35]. Therefore, the enrichment of this pathway at late gestation may be related to a greater concentration of polyamines, and consequently greater placental development during gestation.

**Fig 2:**
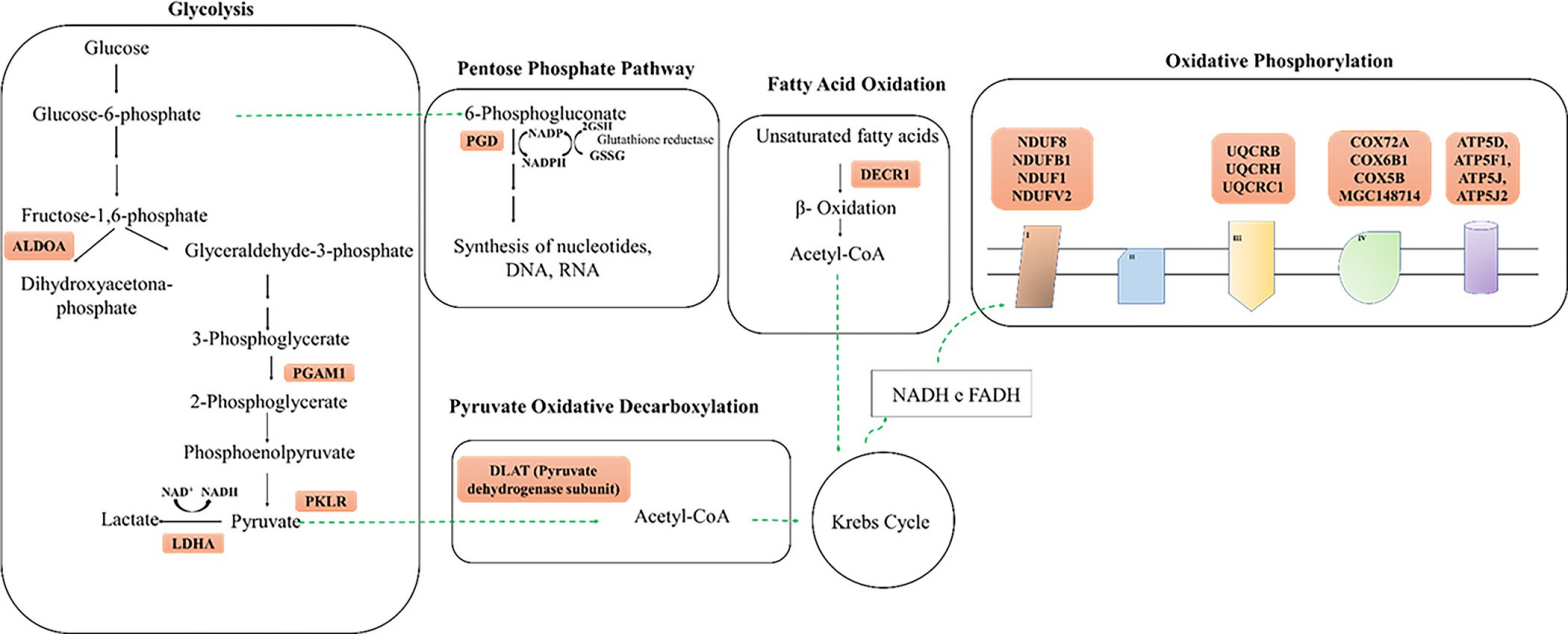
Enriched energy metabolism pathways of the exclusive proteins from treatment PRU. Summary of the energy metabolism pathways influenced by the experimental treatments. Proteins inside the orange square are positively regulated in treatment PRU (post-ruminal releasing of urea) compared to CON. ALDOA: Aldolase; PGAM1: Phosphoglycerate mutase; PKRL: Pyruvate kinase; LDHA: Lactate dehydrogenase; PGD: 6-phosphogluconate dehydrogenase; DLAT: Dihydrolipoamide acetyltransferase; DECR1: 2,4-dienoyl-CoA reductase; NDUFA8, NDUFAB1, NDUFS1 e NDUFV2: Subunits of the complex I of the electron transport chain; UQCRB, UQCRH e UQCRC1: Subunits of the complex III of the electron transport chain; COX72A, COX6B1, COX5B e MGC148714: Subunits of the complex IV of the electron transport chain; ATP5D, ATP5F1, ATP5J, ATP5J2: Subunits of the ATP synthase complex of the electron transport chain.

Evaluating the enriched pathways of the exclusively expressed proteins in PRU cows, we have identified pathways related to carbohydrate digestion, and absorption, glycolysis, pyruvate metabolism, oxidative phosphorylation, pentose phosphate pathway, and biosynthesis of amino acids (Figs 1 and 2). Therefore, our data indicate a change in the hepatic energy balance due to PRU supplementation. These findings can be explained by the improvement in ruminal metabolism by the post-ruminal nitrogen supply, as shown by Oliveira [10] which provided greater input of substrates to enter the metabolic pathways described above. Different isoforms of the protein Na^+^/K^+^ ATPase (ATP1A), responsible for the ATP hydrolysis coupled to the active transport of Na^+^ and K^+^ across the plasma membrane [36] were identified exclusively in PRU cows. Wood [37] observed an increase in the abundance of ATP1A protein in the liver of pregnant cows, suggesting an increase in the metabolic rate during gestation. The action of ATP1A in the liver is related to the active transport of substrates, and the maintenance of ionic homeostasis [38]. Investigating the effects of increasing levels of forages in the diet of growing calves, Wang [39] detected a linear relationship between the abundance of liver ATP1A, and forage inclusion levels, suggesting that the production of short-chain fatty acids in rumen may influence liver ATP1A abundance. Souza [13] observed an increase in volatile fatty acids concentration, and lower pH in rumen when shifting urea release from rumen to the abomasum. This report supports our findings, in which an increase in the abundance of ATP1A in the liver of pregnant cows may be associated with the improvement in ruminal metabolism when supplemented with PRU.

Mitochondria act as a power supplier to the cells through the production of adenosine triphosphate (ATP) coupled to the electron transport via the respiratory chain complexes [40]. Electron transport mechanisms between the mitochondrial complexes may lead to the generation of reactive oxygen species (ROS), since the mitochondria electron transfer in the respiratory chain is not entirely efficient [41]. We observed an exclusive presence of proteins complexes of the electron transport chain; complex I (NDUFA8, NDUFAB1, NDUFS1, and NDUFV2), complex III (UQCRB, UQCRH, and UQCRC1), complex IV (COX72A, COX6B1, COX5B e MGC148714), and ATP synthase (ATP5D, ATP5F1, ATP5J, ATP5J2) in liver of PRU cows (Fig 2), indicating an increase in the oxidative phosphorylation pathway and greater production of ATP in PRU animals. Concomitantly, we found the γ-glutamylcysteine synthase (GCLM) protein exclusively expressed in treatment PRU cows. GCLM is involved in glutathione (GSH) synthesis [42] (Fig 1). The GSH plays an antioxidant role in acting against the reactive oxygen species (ROS) that causes protein degradation [43]. The balance of GSH is closely connected to glucose metabolism through the pentose phosphate pathway, as this pathway generates NADPH used to maintain the reduced levels of GSH [43]. Moreover, the oxidative phosphorylation pathway, which was enriched in PRU treatment, is a potent source of ROS [43]. An imbalance between the increased production of ROS, and the availability of antioxidant defenses needed to reduce ROS accumulation during late gestation may expose cows to increased oxidative stress [44]. The oxidative stress is a significant underlying factor to dysfunctional host immune, and inflammatory responses that can increase the susceptibility of cows to a variety of health disorders, particularly during late gestation [45,46]. Therefore, our result indicates an increase in substrate availability in the liver, which contributed to greater metabolic efficiency in PRU cows. Furthermore, there was enhancement in GSH synthesis in PRU cows that may be reflect a responsive metabolic adaptation to support the increased ROS accumulation and decrease oxidative stress risk generated by increase in the synthesis of ATP in PRU cows.

We have also identified the protein aldolase (ALDOA), to be exclusively expressed in the liver of PRU cows. This protein participates in the last step of the energy-investment phase of glycolysis, catalyzing the conversion of fructose-1,6-bisphosphate into glyceraldehyde 3-phosphate, and dihydroxyacetone phosphate [47] (Fig 2). In the energy-generation phase of glycolysis the exclusively expressed proteins in PRU cows, phosphoglycerate mutase (PGAM1), and pyruvate kinase (PKLR) are involved in the conversion of 3-phosphoglycerate into 2-phosphoglycerate [48], and the conversion phosphoenolpyruvate into pyruvate [49], respectively (Fig 2). As such, our results suggest that the supplementation of PRU sources contribute for the formation of lactate, and acetyl-CoA from pyruvate. Moreover, lactate dehydrogenase (LDHA), also found to be exclusively expressed in PRU cows, catalyzes the conversion of lactate into pyruvate allowing the generation of reducing equivalents (NAD^+^) in anaerobic conditions (Fig 2). Whereas the subunit of the pyruvate dehydrogenase complex, the dihydrolipoamide acetyltransferase (DLAT), exclusively expressed in PRU cows, contribute for the synthesis of acetyl-CoA (Fig 2). In summary, our results indicate that supplementing cows during late gestation with PRU may cause an enhancement in glycolysis pathways, and contribute for the synthesis of acetyl-CoA, leading to the improvement in the liver energy balance.

The 6-phosphogluconate dehydrogenase (PGD) protein, exclusively expressed in PRU cows, play role in the decarboxylation of 6-phosphogluconate to ribulose 5-phosphate, reducing NADP into NADPH, and afterward the ribose-5-phosphate is synthesized (Fig 2). The NADPH is demanded to supply the reduction equivalents of most biosynthetic reactions, such as the fatty acid biosynthesis. Interestingly, the more abundant or exclusively expressed proteins in PRU compared to CON cows, such as methylmalonyl- CoA isomerase (MMUT), 2,4-dienoyl-CoA reductase 1 (DECR1), and branched chain keto acid dehydrogenase E1 subunit alpha (BCKDHA) are involved in energy metabolism, and lipid biosynthesis. The BCKDHA catalyzes the decarboxylation of the α-ketoacids from valine, leucine, and isoleucine, promoting the synthesis propionyl- CoA, whereas the enzyme MMUT regulate the conversion of methylmalonyl-CoA into succinyl- CoA entering the krebs cycle. The DECR1 is involved in the β-oxidation of the unsaturated fatty acids [50]. Taken together, our results suggest that the reduction equivalents synthesized in the pentose phosphate pathways may have been used in β-oxidation, promoting the formation of acetyl-CoA. While the production of ribose-5-phosphate from this pathway contributes to the synthesis of nucleotides, and nucleic acids. Thus, the current study provided evidence that the supplementation of PRU sources is beneficial in liver ATP synthesis, through the enriched pathways of glycolysis, and oxidative phosphorylation.

Protein supplementation has been the focus of the research on pregnant beef cows’ nutrition [51]. When evaluating the partition of crude protein among maternal, and conceptus tissues in ewes during late pregnancy, a moderate net loss of carcass protein was observed over this period [4]. It is important mentioning that amino acid requirements increase in late gestation [1]. Under a scarcity of feedstuffs, maternal skeletal muscle mobilization occurs as an attempt to improve fetal access to amino acids [1]. In addition, part of the amino acids provided by maternal muscle breakdown may be used for the synthesis of milk protein or provide glucose through gluconeogenesis during the first weeks of lactation [52]. However, nutritional strategy that decreases the muscle breakdown in late gestation are recommended to optimize maternal metabolism and to meet fetal requirements. Our results revealed greater urinary level of 3-methyl histidine in CON compared to PRU cows. The 3-methyl histidine is an amino acid derived from the methylation of histidine resulting from the degradation of muscle proteins, and it cannot be reused for protein synthesis, and, consequently, it is excreted in urine [53]. Therefore, the greater urinary 3-methyl histidine in CON cows is an indicative of greater muscle catabolism compared to PRU cows. Therefore, our results suggest that supplementation with PRU reduced muscle catabolism in pregnant cows at late gestation, indicating greater amino acid uptake PRU cows, thus reducing mobilization of maternal skeletal muscle. These findings can be explained by the increase in the ammonia pool for the synthesis of urea in the liver, which in turn generates greater uptake of recycled N by rumen microorganisms [9,10], contributing to the increase in microbial protein, and consequently reducing the demands for muscle protein mobilization. Although phenotypic differences were not observed between the experimental groups, it may cause a long term-effect in cows during the lactating period, and in their progeny performance, as previously described [54], where in the increase in the levels of abomasum infusion of urea contributed to a linear increase in milk protein and urea content that may be benefit calves’ development through a better-quality milk intake. Therefore, supplementation with post-ruminal urea through the diet is an effective way to reduce the amplitude of maternal tissue mobilization during pregnancy and may lead to long-term benefits.

## Conclusion

Shifting urea supply from the rumen to post-ruminal compartments decreases muscle catabolism in cows during late gestation. Our findings indicate that post-ruminal urea supplementation for beef cows at late gestation may improve the energy metabolism to support maternal demands. In addition, the post-ruminal urea release seems to be able to trigger pathways to counterbalance the oxidative stress associated to the increase liver metabolic rate.

## Supporting information

S1 Dataset (XLSX)

## References

[1] Bell AW. Use of ruminants to study regulation of nutrient partitioning during pregnancy and lactation. In: Animal science research and development: moving toward a new century (ed. Ivan M). Minister of Supply and Services, Ottawa, Canada; 1995. pp. 41–62.

[2] Lopes RC, Sampaio CB, Trece AS, Teixeira PD, Gionbelli TRS, Santos LR, et al. Impacts of protein supplementation during late gestation of beef cows on maternal skeletal muscle and liver tissues metabolism. Animal. 2020;14: 1867–75. doi: 10.1017/S1751731120000336 32172711

[3] Bell AW, Burhans WS, Overton TR. Protein nutrition in late pregnancy, maternal protein reserves and lactation performance in dairy cows. Proc Nutr Soc. 2000; 59: 119–26. doi: 10.1017/s0029665100000148 10828181

[4] McNeill DM, Slepetis R, Ehrhardt RA, Smith DM, Bell AW. Protein requirements of sheep in late pregnancy: partitioning of nitrogen between gravid uterus and maternal tissues. J Anim Sci. 1997; 75: 809–16. doi: 10.2527/1997.753809x 9078501

[5] Bell AW, Burhans WS, Overton TR. Protein nutrition in late pregnancy, maternal protein reserves and lactation performance in dairy cows. Proc Nutr Soc. 2000; 59:119–26. doi: 10.1017/s0029665100000148 10828181

[6] Detmann E, Valente EEL, Batista ED, Huhtanen P. An evaluation of the performance and efficiency of nitrogen utilization in cattle fed tropical grass pastures with supplementation. Livest Sci. 2014; 162: 141–53. doi: 10.1016/j.livsci.2014.01.029

[7] Luo QL, Maltby SA, Lobley GE, Calder AG, Lomax MA. The Effect of Amino Acids on the Metabolic Fate of 15NH4Cl in Isolated Sheep Hepatocytes. Eur J Biochem. 1995; 228: 912–7. 7737193

[8] Batista ED, Detmann D, Titigemeyer EC, Valadares Filho SC, Valadares RFD, Prates LL, et al. Effects of varying ruminally undegradable protein supplementation on forage digestion, nitrogen metabolism, and urea kinetics in Nellore cattle fed low-quality tropical forage. J Anim Sci. 2016; 94: 201–16. doi: 10.2527/jas.2015-9493 26812327

[9] Carvalho PCd, Doelman J, Martín-Tereso J. Post-ruminal non-protein nitrogen supplementation as a strategy to improve fibre digestion and N efficiency in the ruminant. J Anim Physiol Anim Nutr. 2020; 104: 64–75. doi: 10.1111/jpn.13233 31674078

[10] Oliveira CVR, Silva TE, Batista ED, Rennó LN, Silva FF, De Carvalho IPC, et al. Urea supplementation in rumen and post-rumen for cattle fed a low-quality tropical forage. Br J Nutr. 2020; 124: 1166–78. doi: 10.1017/S0007114520002251 32580810

[11] Costa T, Gionbelli M, Duarte M. Fetal programming in ruminant animals: understanding the skeletal muscle development to improve the quality of meat. Anim Front. 2021; 11: 66–73. doi: 10.1093/af/vfab061 34934531 PMC8683153

[12] Moreira GM, Aguiar GL, Meneses JAM, Luz MH da, Monteiro MGBB, Lara L, et al. The course of pregnancy changes general metabolism and affects ruminal epithelium activity pattern in Zebu beef heifers. Livest Sci. 2021; 248: 104496. doi: 10.1016/j.livsci.2021.104496

[13] Souza MG, Reis IA, Carvalho IPCd, Porcionato MADF, Prados LF, Granja-Salcedo YT, et al. Effects of post-ruminal urea supplementation during the seasonal period on performance and rumen microbiome of rearing grazing nellore cattle. Animals. 2022; 12: 3463. doi: 10.3390/ani12243463 36552384 PMC9774649

[14] Valadares Filho SC, Costa e Silva LF, Gionbelli MP, Rotta PP, Marcondes MI, Chizzotti ML, et al. BR-CORTE 3.0. Nutrient Requirements of Zebu and Crossbred Cattle Third Edition, 3rd ed. Vicosa. 2016. doi: 10.5935/978–85–8179–111–1.2016B002

[15] Mølgaard L, Damgaard BM, Bjerre-Harpøth V, Herskin MS. Effects of percutaneous needle liver biopsy on dairy cow behaviour. Res Vet Sci. 2012; 93: 1248–1254. doi: 10.1016/j.rvsc.2012.04.001 22542802

[16] AOAC, editor. Oficial methods of analysis. 18th ed. Gaithersburg: AOAC International; 2005.

[17] Detmann E, Souza MA, Valadares Filho SC, Queiroz AC, Berchielli TT, Saliba EOS, et al. Métodos para análise de alimentos. 1st edition. Suprema, Visconde do Rio Branco, Minas Gerais, Brazil. 2012.

[18] Detmann E, Valadares Filho S.C. On the estimation of non-fibrous carbohydrates in feeds and diets. Arq. Bras. Med. Vet. Zootec; 2010; 62: 980–4. doi: 10.1590/S010209352010000400030

[19] Casali AO, Detmann E, Valadares Filho SC, Pereira JC, Cunha M, Detmann KSC, et al. Estimation of fibrous compounds contents in ruminant feeds with bags made from different textiles. Rev Bras Zootec. 2008; 38: 130–8. doi: 10.1590/S1516-35982009000100017

[20] Robinson MD, Oshlack A. A scaling normalization method for differential expression analysis of RNA-seq data. Genoma Biol. 2010; 11: R25. doi: 10.1186/gb-2010-11-3-r25 20196867 PMC2864565

[21] Osborne JW, Overbay A. The power of outliers (and why researchers should ALWAYS check for them). Practical Assessment, Research, and Evaluation. 2004; 9: 1–8. doi: 10.7275/qf69-7k43

[22] Koziol JA, Griffin NM, Long F, Li Y, Latterich M, Schnitzer JE. On protein abundance distributions in complex mixtures. Proteome Sci. 2013; 11: 3–9. doi: 10.1186/1477-5956-11-5 23360617 PMC3599228

[23] Storey JD. A direct approach to false discovery rates. J R Stat Soc Ser B Stat Methodol. 2002; 64: 479–98. doi: 10.1111/1467-9868.00346

[24] Storey JD, Bass AJ, Dabney A, Robinson D. qvalue: Q-value estimation for false discovery rate control. R package version 2.28.0. 2022. http://github.com/jdstorey/qvalue. [accessed on 3 Sept 2022].

[25] R Core Team (2021). R: A language and environment for statistical computing. R Foundation for Statistical Computing, Vienna, Austria. https://www.R-project.org/.

[26] Posit team (2023). RStudio: Integrated Development Environment for R. Posit Software, PBC, Boston, MA. http://www.posit.co/.

[27] Benjamini Y, Drai D, Elmer G, Kafkafi N, Golani I. Controlling the false discovery rate in behavior genetics research. Behav Brain Res. 2001; 125: 279–84. doi: 10.1016/s0166-4328(01)00297-2 11682119

[28] Szklarczyk D, Morris JH, Cook H, Kuhn M, Wyder S, Simonovic M, et al. The STRING database in 2017: Quality-controlled protein-protein association networks, made broadly accessible. Nucleic Acids Res. 2017; 45: D362–D368. doi: 10.1093/nar/gkw937 27924014 PMC5210637

[29] Mato JM, Alvarez L, Ortiz P, Pajares MA. S-adenosylmethionine synthesis: Molecular mechanisms and clinical implications. Pharmacol Ther. 1997; 73: 265–280. doi: 10.1016/s0163-7258(96)00197-0 9175157

[30] Bae DH, Lane DJR, Siafakas AR, Sutak R, Paluncic J, Huang MLH, et al. Acireductone dioxygenase 1 (ADI1) is regulated by cellular iron by a mechanism involving the iron chaperone, PCBP1, with PCBP2 acting as a potential co-chaperone. BBA—Mol Basis Dis. 2020; 1866: 165844. doi: 10.1016/j.bbadis.2020.165844 32480040

[31] Fozard JR, Part M-L, Prakash NJ, Grove J, Schechter PJ, Sjoerdsma A, et al. L-Ornithine Decarboxylase: an Essential Role in Early Mammalian Embryogenesis. Science 1980; 208: 505–08. doi: 10.1126/science.6768132 6768132

[32] Avila MA, García-Trevijano ER, Lu SC, Corrales FJ, Mato JM. Methylthioadenosine. Int J Biochem Cell Biol. 2004; 36: 2125–30. doi: 10.1016/j.biocel.2003.11.016 15313459

[33] Kwon H, Wu G, Bazer FW, Spencer TE. Developmental Changes in Polyamine Levels and Synthesis in the Ovine Conceptus. Biol Reprod. 2003; 69: 1626–34. doi: 10.1095/biolreprod.103.019067 12855596

[34] Reynolds LP, Redmer DA. Angiogenesis in the placenta. Biol. Reprod. 2001; 64: 1033–1040. doi: 10.1095/biolreprod64.4.1033 11259247

[35] Sladek SM, Magness RR, Conrad KP. Nitric oxide and pregnancy. Am. J. Physiol.—Regul. Integr. Comp. Physiol. 1997; 272: R441–R463. doi: 10.1152/ajpregu.1997.272.2.R441 9124465

[36] Herrera VLM, Emanuel JR, Ruiz-Opazo N, Levenson R, Nadal-Ginard B. Three differentially expressed Na, K-ATPase α subunit isoforms: Structural and functional implications. J Cell Biol. 1987; 105: 1855–65. doi: 10.1083/jcb.105.4.1855 2822726 PMC2114652

[37] Wood KM, Awda BJ, Fitzsimmons C, Miller SP, Mcbride BW, Swanson KC. Influence of pregnancy in mid-to-late gestation on circulating metabolites, visceral organ mass, and abundance of proteins relating to energy metabolism in mature beef cows. J Anim Sci. 2013; 91:5775–84. doi: 10.2527/jas.2013-6589 24146152

[38] McBride BW, Kelly JM. Energy cost of absorption and metabolism in the ruminant gastrointestinal tract and liver: a review. J Anim Sci. 1990; 68: 4445. doi: 10.2527/1990.6892997x 2170320

[39] Wang YJ, Holligan S, Salim H, Fan MZ, McBride BW, Swanson KC. Effect of dietary crude protein level on visceral organ mass, cellularity, and the protein expression of ATP synthase, Na+/K+-ATPase, proliferating cell nuclear antigen and ubiquitin in feedlot steers. Can J Anim Sci. 2009; 89: 493. doi: 10.4141/CJAS08131

[40] Wilson DF. Oxidative phosphorylation: regulation and role in cellular and tissue metabolism. JPhysiol. 2017; 595(23): 7023–7038. doi: 10.1113/JP273839 29023737 PMC5709332

[41] Castro JP, Jung T, Grune T, Almeida H. Actin carbonylation: from cell dysfunction to organism disorder. J. Proteomics. 2023; 92: 171–180. doi: 10.1016/j.jprot.2013.05.006 23684956

[42] Richman PG, Orlowski M, Meister A. Inhibition of γ glutamylcysteine synthetase by L methionine S sulfoximine. J Biol Chem. 1973; 248: 6684–90. doi: 10.1016/s0021-9258(19)43407-8 4147652

[43] Holmström KM, Finkel T. Cellular mechanisms and physiological consequences of redox-dependent signalling. Nat Rev Mol Cell Biol. 2014; 15: 411–21. doi: 10.1038/nrm3801 24854789

[44] Sordillo LM, Aitken SL. Impact of oxidative stress on the health and immune function of dairy cattle. Vet Immunol Immunopathol. 2009; 128: 104–09. doi: 10.1016/j.vetimm.2008.10.305 19027173

[45] Castillo C, Hernandez J, Bravo A, Lopez-Alonso M, Pereira V, Benedito JL. Oxidative status during late pregnancy and early lactation in dairy cows. Vet J. 2005; 169: 286–92. doi: 10.1016/j.tvjl.2004.02.001 15727923

[46] Sordillo LM. Factors affecting mammary gland immunity and mastitis susceptibility. Livest Prod Sci. 2005; 98: 89–99. doi: 10.1016/j.livprodsci.2005.10.017

[47] Neuzil J, Danielson H, Welch GR, Ovádi J. Cooperative effect of fructose bisphosphate and glyceraldehyde-3-phosphate dehydrogenase on aldolase action. Biochim Biophys Acta (BBA)/Protein Struct Mol. 1990; 1037: 307–12. doi: 10.1016/0167-4838(90)90030-j 2106914

[48] Kun E. Conversion of 3-Phosphoglycerate to Phosphoenolpyruvate by Tissue Homogenates. Exp Biol Med. 1950; 75: 68–71. doi: 10.3181/00379727-75-18103 14797739

[49] Gray LR, Tompkins SC, Taylor EB. Regulation of pyruvate metabolism and human disease. Cell Mol Life Sci. 2014; 71: 2577–604. doi: 10.1007/s00018-013-1539-2 24363178 PMC4059968

[50] Ramírez O, Quintanilla R, Varona L, Gallardo D, Díaz I, Pena RN, et al. DECR1 and ME1 genotypes are associated with lipid composition traits in Duroc pigs. J Anim Breed Genet. 2014; 131: 46–52. doi: 10.1111/jbg.12035 25099788

[51] Barcelos SS, Nascimento KB, Silva TM, Mezzomo R, Alves KS, Duarte MD, et al. The Effects of Prenatal Diet on Calf Performance and Perspectivesfor Fetal Programming Studies: A Meta-Analytical Investigation. Animals. 2022; 12: 2145. doi: 10.3390/ani12162145 36009734 PMC9404886

[52] Blum JW, Reding T, Jans F, Wanner M, Zemp M, Bachmann K. Variations of 3-Methylhistidine in Blood of Dairy Cows. Journal of Dairy Science. 1985; 68(10): 2580–87. doi: 10.3168/jds.S0022-0302(85)81140-1 4067036

[53] Waterlow JC. Protein turnover. 2nd ed. Wallingford: CABI Publishing, 2006.

[54] Nichols K, Lippens L, Seymour D, Rauch R, Martín-Tereso J. Exploring the threshold of non-protein nitrogen in dairy cattle diets by postruminal urea delivery. Journal of Dairy Science. 2021;104 (suppl. 1)100.

